# Lipid accumulation mechanism of *Amphora coffeaeformis* under nitrogen deprivation and its application as a feed additive in *Carassius auratus* aquaculture

**DOI:** 10.1186/s13068-023-02436-7

**Published:** 2023-12-06

**Authors:** Yulin Cui, Kang Wang, Xiuzhi Zhou, Chunxiao Meng, Zhengquan Gao

**Affiliations:** 1 Binzhou Medical University, No. 346, Guanhai Road, Laishan District, Yantai, 256603 Shandong Province China; 2 Key Laboratory of Coastal Biology and Biological Resource Utilization, Yantai Institute of Coastal Zone Research, Chinese Academy of Sciences, Yantai, 264003 Shandong China; 3 University of Chinese Academy of Sciences, Beijing, Beijing, 101418 China

**Keywords:** *Amphora coffeaeformis*, Nitrogen deprivation, Lipid accumulation, RNA-seq, TAG, Crucian carp, Fatty acids

## Abstract

**Background:**

*Amphora coffeaeformis*, a unicellular diatom, can significantly accumulate lipids under nitrogen (N) limitation. However, the molecular mechanism underlying lipid accumulation in *A. coffeaeformis* remains unknown and its application development is lagging.

**Results:**

This work analyzed the lipid composition of *A. coffeaeformis* under N deprivation and investigated its mechanism underlying lipid accumulation using RNA-seq. The results showed that the total lipid content of *A. coffeaeformis* increased from 28.22 to 44.05% after 5 days of N deprivation, while the neutral lipid triacylglycerol (TAG) content increased from 10.41 to 25.21%. The transcriptional profile showed that N deprivation induced wide-ranging reprogramming of regulation and that most physiological activities were repressed, while the upregulation of glycerol-3-phosphate acyltransferase directly determined TAG accumulation. Moreover, we explored the effect of *A. coffeaeformis* as a food additive on the lipid composition of crucian carp. The results showed that the contents of unsaturated fatty acids in the meat of fish supplemented with *A. coffeaeformis* were significantly increased, indicating its potential application in animal nutrition for improving meat quality indicators.

**Conclusion:**

The findings shed light on the molecular mechanisms of neutral lipid accumulation and revealed the key genes involved in lipid metabolism in *A. coffeaeformis.* Moreover, we also confirmed that *A. coffeaeformis* can be used as feed additive for improving the lipid composition of crucian carp meat, which provided evidence for the biotechnology application of this high-oil microalgae.

**Supplementary Information:**

The online version contains supplementary material available at 10.1186/s13068-023-02436-7.

## Introduction

Microalgae are one of the potential materials for the production of food, feed, biofuel, and other high-value compounds due to their high carbohydrate and fat content, fast growth rate, and high resistance to environmental stress [1, 2]. Currently, only a limited number of algal strains are being utilized for biomass production across various domains [2]. The industrialization of microalgae depends on developing new strains, particularly marine species.

Diatoms account for 40% of the primary productivity of marine ecosystems. Some species, such as *Phaeodactylum tricornutum*, *Amphora* sp., and *Nitzschia* sp., are known to accumulate neutral lipids [3]. Furthermore, diatoms are metabolically versatile, as they can synthesize and accumulate wide ranges of valuable compounds, such as polyunsaturated fatty acids (PUFAs) [4, 5]. Therefore, they have attracted both biological and medical attention. Generally, oil levels of 20–50% of dry weight (DW) are common in microalgae, which can reach up to 70% of dry mass when cells are subject to physiological stress conditions or unfavorable environments, such as nutrient limitation or photo-oxidative stress [6, 7]. Nutrient availability is of considerable importance for the growth and primary production of microalgae [8]. Typical nutrient limitation in nature affects the supply of nitrogen (N), phosphate, and/or silicate [9]. Nitrogen is the main nutrient element that affects the growth and oil content of microalgae [10, 11]. However, although microalgae can attain the maximum total biomass under high nitrogen concentrations, the total lipid content decreases [12]. In contrast, N limitation can lead to a decrease in protein content and a relative increase in carbohydrate and/or lipid storage, and can also result in a reduction in growth rate and photosynthetic efficiency, thereby affecting the final biomass of microalgae [13, 14]. In addition, nitrogen is also necessary for some microalgae to synthesize unsaturated fatty acids. Insufficient nitrogen not only increases the content of saturated fatty acids in the algal cells, but also reduces the content of PUFAs [15]. Yang et al. [3] found that the content of polyunsaturated fatty acids in *P. tricornutum* decreased when the nitrogen source was insufficient. The main reason may be that the lack of nitrogen reduces the synthesis of amino acids, which in turn reduces the production of pigment complex (rich in protein), which leads to a reduction in the demand for phospholipids and glycerol lipids by algal cells [16]. However, although previous studies have successfully demonstrated the role of N limitation in lipid accumulation in diatoms [17], the underlying metabolic pathways remain largely variable between different strains.

In this study, we investigated the response of a unicellular diatom, *A. coffeaeformis*, to N deprivation conditions and its lipid accumulation mechanisms with a view to developing its potential for commercial application. In addition, we also confirmed that *A. coffeaeformis*, as a feed additive, significantly improved the lipid composition of crucian carp meat, which provided evidence for the biotechnology application of this high-oil microalgae.

## Results and discussion

### Effect of N deprivation conditions on *A. coffeaeformis*

#### N deprivation affected the growth and physiological status of *A. coffeaeformis*

**Fig. 1:**
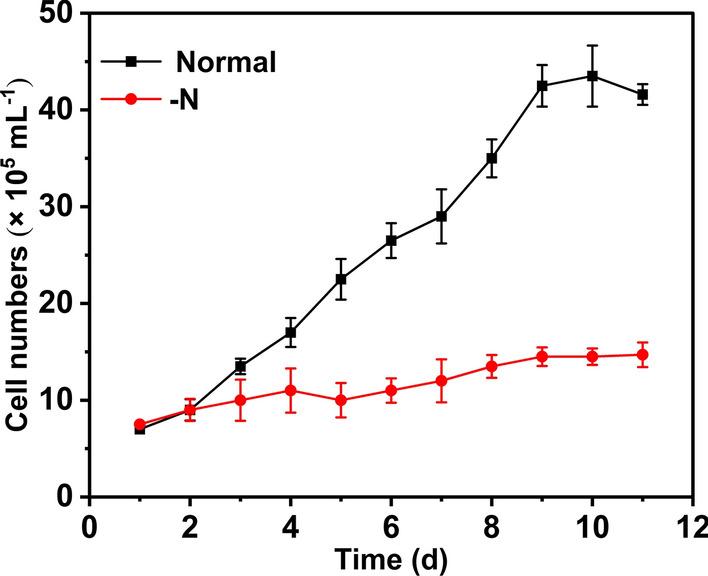
Growth curve of *A. coffeaeformis* under N-deprived and normal conditions. Cells were cultured at 30 ℃ with 12 h/12 h light cycle and 100–110 μ mol/m^2^ s^−1^ light intensity. − N, cells were cultured at N-deprived f/2 medium; normal, cells were cultured at normal f/2 medium

To monitor growth, single cells of *A. coffeaeformis* were separated in three Petri dishes and observed using an inverted microscope every 24 h. The growth curve is shown in Fig. 1. The growth rate was 0.22 per day, the division was 0.32 per day, and the resulted doubling time was 3.15 days. The growth rate of *A. coffeaeformis* is comparable with those of the other known *Amphora* algae [18], indicating that the conditions in this experiment were suitable for this species.

**Fig. 2:**
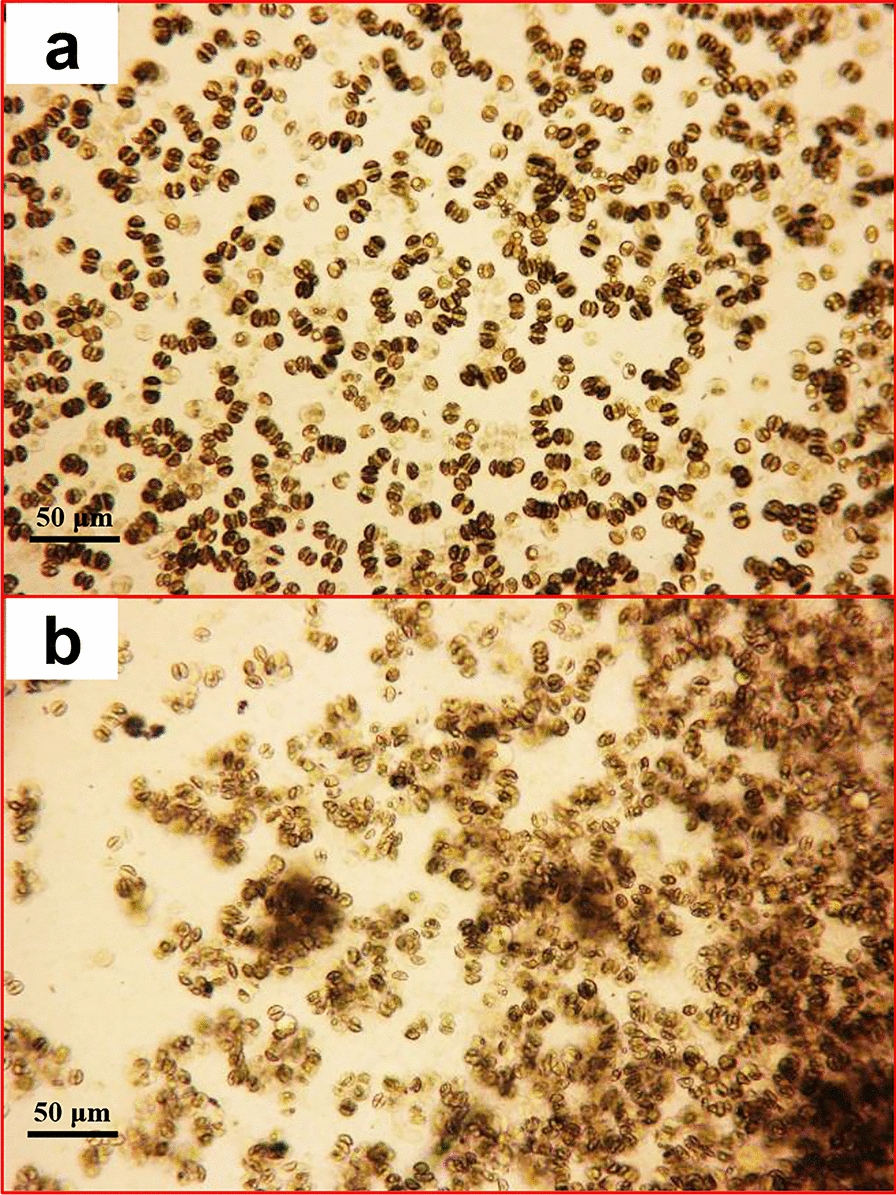
Attachment status of *A. coffeaeformis* cultured on normal medium (**a**) or under N-deprived medium (**b**) for 7 days, 10 × 10 magnified

As previously reported in other oleaginous microalgae [19], the lack of nitrogen inhibits growth and significantly shortens the time required for *A. coffeaeformis* in the stable phase to enter the recession stage. The growth rate of *A. coffeaeformis* in f/2-N medium was 0.06 per day and the doubling time extended to 11.67 days (Fig. 1). For *A. coffeaeformis* culture in the stable phase, N deprivation restrained growth and attachment status. As shown in Fig. 2, the *A. coffeaeformis* in normal f/2 medium was uniformly attached to the bottom of Petri dishes; in contrast, some cells fell off the substratum after 5 days of N deprivation. The stable phase of *A. coffeaeformis* under normal conditions was maintained for more than 20 days, following which, some cell clusters fell off, were suspended in the medium, and died (recession stage). However, N deprivation shortened the stable phase of *A. coffeaeformis* to 7–8 days and the recession stage to 10 days.

**Fig. 3:**
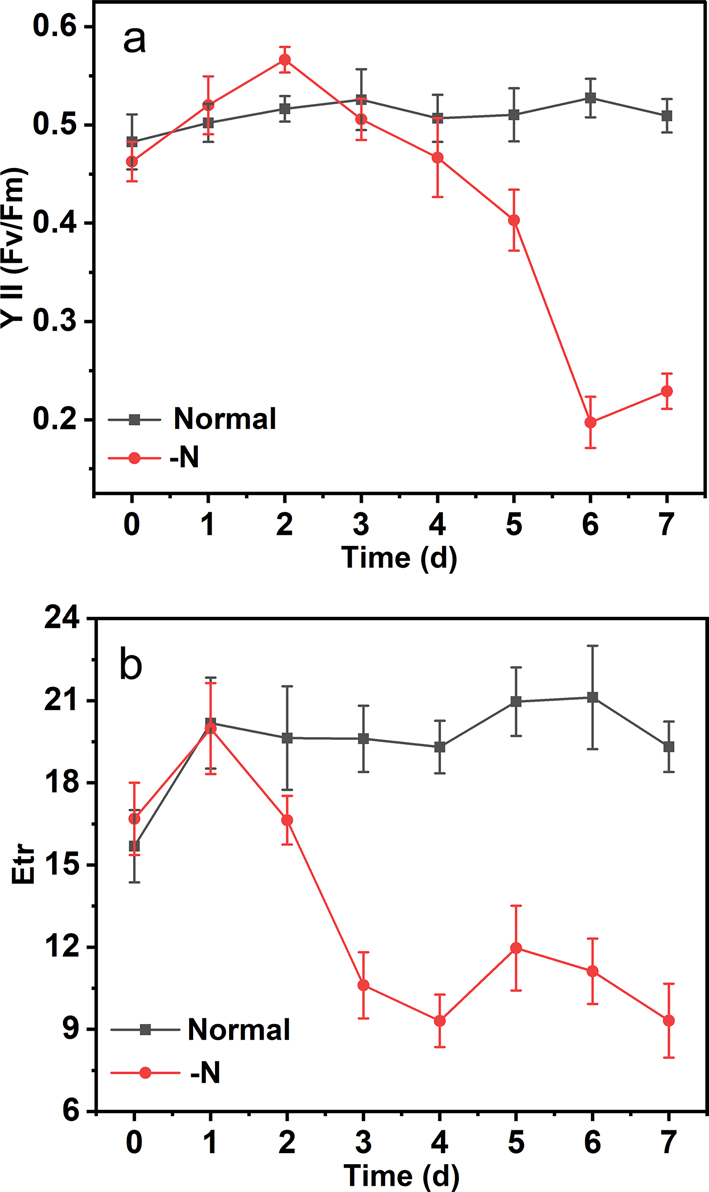
Effect of N deprivation on the physiological status of *A. coffeaeformis*. **a** The maximum PSII photochemical efficiency (Fv/Fm). **b** Electron transfer rate (Etr). Each value represents mean ± SD (*n* = 3)

The response of the photosynthetic performance to growth in nitrogen-rich and nitrogen-free media is particularly important for improving our understanding regarding the metabolic and physiological differences in diatoms under N deprivation [15]. Photosynthesis performance parameters can directly reflect the growth status of microalgae, providing detailed information regarding the cellular physiology of the algae under N deprivation [20]. Therefore, the photosynthetic activity was examined during N deprivation to monitor the physiological status of *A. coffeaeformis*. The maximum PSII photochemical efficiency Fv/Fm and Etr characterize the physiological response of microalgal cells to changes in environmental conditions, such as nutrient starvation or photoinhibition [15]. In this study, Fv/Fm and Etr declined in N-deprived medium (Fig. 3). Especially the character YII of *A. coffeaeformis* after 6 days’ N deprivation declined dramatically (0.46 to 0.19) (Fig. 3a). Obviously, the physiological activity of *A. coffeaeformis* under N deprivation was inhibited owing to inhibition of photosynthetic activity.

#### N deprivation promoted lipid accumulation in *A. coffeaeformis*

**Fig. 4:**
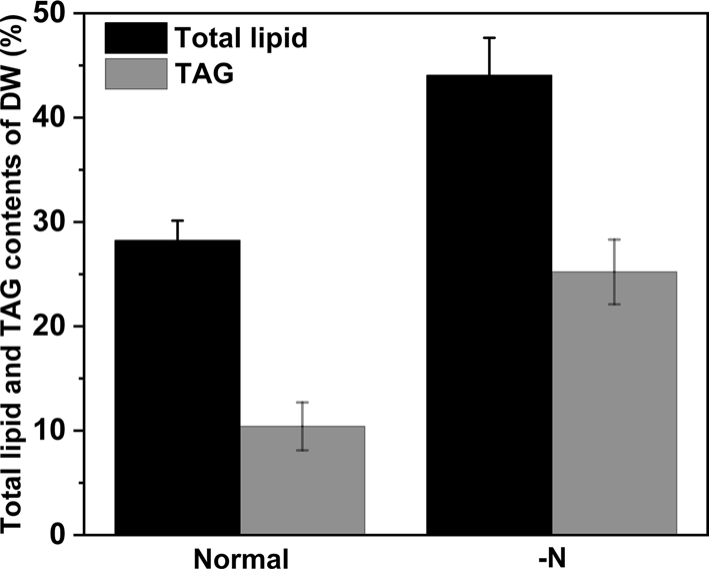
Total lipid and TAG contents under normal and N-deprived conditions. Each value represents mean ± SD (*n* = 3)

The accumulation of lipids in microalgae under N deficiency has been studied for decades. Studies have shown that the growth of microalgae (i.e., synthesis of protein and nucleic acids) is limited under N deficiency, although the process of carbon assimilation is always ongoing, which channels more carbon into lipid metabolism [13, 15]. As an important component of lipids, TAGs do not contain nitrogen. Therefore, despite the lack of nitrogen in microalgae under conditions supporting ongoing carbon assimilation, TAGs can accumulate rapidly in cells. Converti et al. [21] showed that when the nitrogen source in the culture medium was reduced by 75%, the total lipid content in *Nannochloropsis oculata* increased from 7.9 to 15.31%, while the total lipid content of *Chlorella vulgaris* increased from 5.9 to 16.41%. In the present study, the total lipid content of *A. coffeaeformis* in normal f/2 medium was 28.22% (TL/DW), which increased to 44.05% after 5 days of N deprivation, while the neutral lipid TAG content increased from 10.41 to 25.21% (TAG/DW) (Fig. 4). The lipid content in *A. coffeaeformis* was as high as that reported in other *Amphora* species.

The distinct difference between the fatty acid compositions of *A. coffeaeformis* in f/2 medium and under N deprivation was not observed (Table 1). Surprisingly, the fatty acid compositions of TAG in N-deprived *A. coffeaeformis* differed considerably from that of the control (Table 2). The saturated fatty acid contents increased dramatically from 57.94 to 81.21%, especially that of the long-chain fatty acids C16:0, C18:0, and C20:0. Furthermore, the content of the unsaturated fatty acids decreased. These results indicated that the key enzymes in the TAG synthesis pathway may facilitate the metabolism of saturated fatty acids and as a result, the unsaturated fatty acids flowed to the other lipids, with the exception of TAG.

**Table 1:** Fatty acid composition (total FA %) of *A. coffeaeformis* following N deprivation

Fatty acids	Control	− N (5 d)
C12:0	0.17	0.165
C13:0	0.1	0.09
C14:0	10.02	10.04
C14:1	0.15	0.13
C14:1t	2.17	2.13
C14:1t	4.57	4.31
C15:0	2.81	2.66
C16:0	23.48	23.41
C16:1	24.28	25.31
C16:1t	0.62	0.61
C16:2	1.24	1.32
C16:3	2.27	2.32
C18:0	1.57	1.245
C18:1	2.34	2.32
C18:1t	1.3	1.175
C18:2	2.76	2.715
C18:3	0.06	0.06
C19:1	0.76	0.79
C18:3	0.15	0.15
C20:0	0.07	0.07
C20:3	0.24	0.25
C20:4	8.06	7.95
C20:5	8.67	8.775
C24:0	0.97	0.96
C24:1	0.56	0.06

**Table 2:** Fatty acid composition (total FA %) in TAGs with significant changes following N deprivation

Fatty acids	Normal	− N (5 d)
14:0	7.52	4.99
16:0	30.72	33.08
16:1	23.17	7.3
16:1t	2.77	5.51
18:0	10.29	29.14
18:1	3.92	6.81
18:1t	1.97	1.47
18:2	1.82	5
18:3	0.34	4.56
20:0	1.06	3.66

### Mechanism of lipid accumulation in *A. coffeaeformis* under N deprivation

#### High-throughput analysis and DEGs in response to N-deprived conditions

**Fig. 5:**
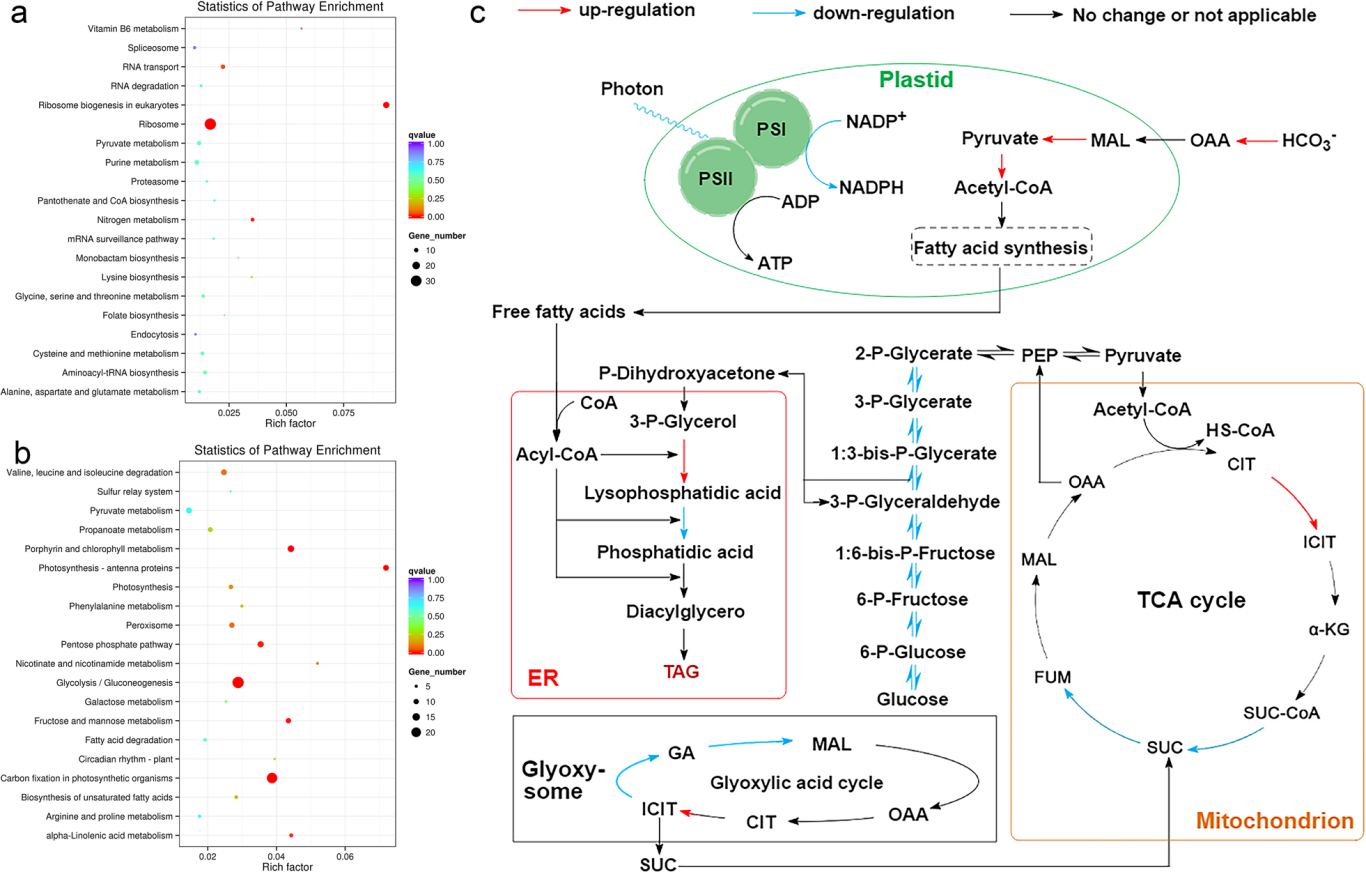
High-throughput analysis and DEGs in response to N-deprived conditions. **a** KEGG enrichment analyses of DEGs of *A. coffeaeformis*. a, KEGG pathway analysis of upregulated DEGs. **b** KEGG pathway analysis of downregulated DEGs. **c** Proposed general transcriptional changes of *A. coffeaeformis* under N deprivation. Schematic diagram showing the putative localization of central metabolic pathways of *A. coffeaeformis*

To obtain in-depth knowledge regarding the mechanisms, comprehensive analyses such as analysis of gene expression should be used. Therefore, the comprehensive transcriptome data of *A. coffeaeformis* under N deprivation were compared with that under normal conditions. The data showed that 591 genes were upregulated and 1,021 genes were downregulated (Additional file 1: Fig. S1), implying induced extensive regulatory reprogramming. KEGG analysis for DEGs classified them as being associated with different functional categories, in which multiple genes were significantly upregulated or downregulated, suggesting possible mechanisms involved in the response to N deprivation. For example, genes mediating the metabolism of nitrogenous compounds and ribosome biogenesis were upregulated, while genes encoding enzymes required for photosynthesis, biosynthesis of unsaturated fatty acids, carbon fixation, glycolysis, and gluconeogenesis were generally downregulated under N deprivation (Fig. 5a, b; Additional file 1: Fig. S2). It can be speculated that certain biochemical processes might be involved in adaptation to the lack of nitrogen. Genes with log_2_-fold differences in transcripts (Additional file 1: Table S1) between the control group and N-deprived *A. coffeaeformis* were considered global changes in major categories of genes involved in various pathways, which reflected general transcriptional responses to N deprivation [17], are shown in Fig. 5c.

#### Transcript changes involving different metabolic pathways were investigated

The main effect of nitrogen deprivation is the reduction in nitrogen availability and eventually cause the synthesis of nitrogenous compounds hindered [17, 22]. Therefore, there is no doubt that the transcription levels of genes in *A. coffeaeformis* involved in nitrogen assimilation and metabolism were significantly affected under nitrogen deprivation (Additional file 1: Table S1). For example, the transcripts encoding three nitrate reductase (NADH-nitrate reductase, EC 1.7.1.1; assimilatory NAD(P)H-nitrate reductase, EC 1.7.1.2; assimilatory NADPH-nitrate reductase, EC 1.7.1.3) that catalyzes the reaction of nitrate to nitrite were increased with 3.4-fold changes. We also observed that the transcript of glutamine synthase (EC 6.3.1.2), which plays an important role in the effective utilization of nitrogen sources and nitrogen metabolism, increased significantly by 2.1-fold. Meanwhile, significant increases were also found in three transcripts encoding ammonium transporters present in microalgae, which are known to be activated by N deprivation, thereby transporting ammonium ions across the cell membrane [17, 23]. It can be speculated that N deficiency induces a steady-state response, including the activation of the glutamine synthesis pathway and the increase in the ability of cells to utilize trace nitrogen resources, as well as the possible redistribution of intracellular nitrogen [17, 24].

For photosynthetic cells, inhibition or stop of photosynthesis means stagnation of cell growth or death. In fact, the transcripts encoding proteins related to photosynthesis were investigated under nitrogen deficiency, and the levels of most transcripts were decreased (Additional file 1: Table S1). Notably, ferredoxin-NADP^+^ reductase (EC 1.18.1.2), an enzyme that catalyzes the last electron transfer (from photosystem I to NADPH) during photosynthesis, was observed the decreased transcription levels (4.8-fold). The decreased transcripts of genes encoding photosynthetic proteins were also consistent with expectations [25, 26].

Pyruvate-phosphate dikinase (PPDK, EC 2.7.9.1) catalyzes the synthesis of PEP from pyruvate. The transcripts of two PPDK genes in *A. coffeaeformis* under N deprivation were significantly decreased (1.7-fold and 2.2-fold, respectively). It can be predicted that the reduction of PPDK level reduces the consumption of pyruvate, thereby enables more pyruvate to synthesize acyl-CoA, the precursor of fatty acid, while the excessively expressed PEPCs can use the available PEP for carbon fixation. Moreover, PPDK is also involved in PPDK-mediated gluconeogenesis [27], which indicates that the decreases of PPDKs expression may inhibit the activation of gluconeogenesis. Notably, the significant increased transcripts of a gene encoding NADP^+^-malic enzyme (ME, EC 1.1.1.40) was also observed. It is speculated that this gene may be involved in the carbon fixation pathway in *A. coffeaeformis*, and is localized in the chloroplast. ME catalyzes the irreversible decarboxylation of malate to pyruvate in photosynthetic cells with the formation of NADPH from NADP^+^, which is the rate-limiting step of fatty acid biosynthesis [17]. In fact, many previous reports have confirmed the promoting effect of ME overexpression on fatty acid synthesis. For example, the overexpression of endogenous NADP-dependent ME in *N. salina* enhanced the lipid production, and the report also analyzed the total carbon concentration and NADPH/NADP^+^ ratio, which were found to be enhanced in the transformants [28]. Hence, the upregulation of ME in *A. coffeaeformis* under N deprivation may increase the NADPH production, thereby providing both reducing power and cofactors for reactions catalyzed by enzymes involved in fatty acid synthesis such as ACCase, fatty acid synthase, and eventually leading to increases in TAG accumulation [17].

#### Increased GPAT levels promoted TAG biosynthesis

To further determine the precise mechanism via which the microalga upregulated gene expression under N deprivation, genes involved in TAG and fatty acid biosynthesis were studied. Notably, N deprivation increased the transcript levels of genes associated with TAG biosynthesis. In particular, the expression of glycerol-3-phosphate *O*-acyltransferase (GPAT, EC 2.3.1.15) that catalyze the first committed step of TAG biosynthesis increased (1.2-fold); thus, the increase in their mRNA abundance under N deprivation may have increased TAG levels.

However, transcripts of the gene encoding lysophosphatidic acid-acyltransferase (LPAAT, EC 2.3.1.51) that catalyzes the second step of TAG biosynthesis decreased (1.2-fold). Interestingly, the significant differences in transcripts encoding diacylglycerol acyltransferase (DGAT, EC 2.3.1.20) that catalyzes the final committed step of TAG biosynthesis, were not observed, and the mRNA levels of another enzyme, responsible for the last step of TAG biosynthesis, phospholipid:diacylglycerol acyltransferase (PDAT, EC 2.3.1.158), decreased 1.8-fold. These findings suggest that the overexpression of GPAT seems to be a key factor in TAG biosynthesis of *A. coffeaeformis*. In fact, some previous reports also showed the same phenomenon. For example, only transcripts of GPAT1 and GPAT2 were increased among fatty acid and TAG biosynthesis genes under TAG accumulation conditions in *Cyanidioschyzon merolae* [29]. Similarly, overexpression of GPAT1 and GPAT2 in *C. merolae* resulted in up to a 56.1-fold increase in seed oil content [30].

To further confirm the expression levels of key genes involved in TAG biosynthesis pathway, quantitative real-time PCR was carried out, and *gpat*, *lpaat*, and *pdat* were selected as target genes, respectively. As expected, transcript levels of *gpat* and *lpaat* were decreased under nitrogen deprivation conditions, while transcript levels of the *pdat* were slightly increased (Table 3). Taken together, the reaction catalyzed by the ER-localized GPAT is a rate-limiting step for TAG synthesis in *A. coffeaeformis*, and would be a potential target for improvement of TAG productivity in microalgae.

**Table 3:** qRT-PCR of differentially expressed genes following N deprivation

Gene	Location	Forward (5′–3′)	Reverse (5′–3′)	Log_2_FC (qRT-PCR)	Log_2_FC (RNA-seq)
*gpat*	Cluster-30447.20498	ACCTTGAAGGTT	ACGGTATTGCTC	1.248	1.241
		TCCATGGCCTT	GGGCATACTTT		
*lpaat*	Cluster-30447.18970	TTTGTTGCCTCG	TTGCAGGATACC	− 1.475	− 1.184
		GCCTGGCA	AAACATGACGG		
*pdat*	Cluster-30447.20296	TTATCGCTCCTTC	GCATCCACAGG	− 1.222	− 1.768
		CATTCTTTCG	AGAAACCATC		

### Application development of *A. coffeaeformis* as feed additive in aquaculture

#### Effect of silicate concentration on the growth of *A. coffeaeformis*

**Fig. 6:**
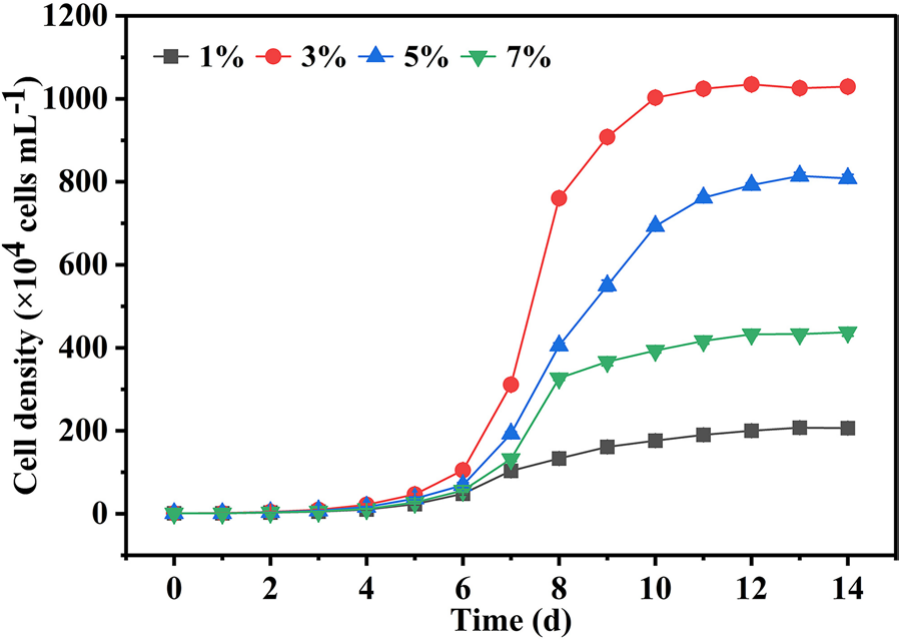
Effect of different initial silicate (1, 3, 5, and 7%) on the growth of *A. coffeaeformis* in a tubular photobioreactor. Each value represents mean ± SD (*n* = 3)

To obtain the best biomass production, the effects of silicate on the growth of *A. coffeaeformis* in a tubular photobioreactor were also investigated. It is well known that initial silicate can influence the growth and biomass production of diatoms [31]. A suitable initial silicate concentration can help us get more production of *A. coffeaeformis*. If the level of initial silicate concentration is too low, the growth of *A. coffeaeformis* will be in the period of lag phase for a long time. On the other hand, a high level of initial silicate will also inhibit the growth of diatoms. In our research, the highest cell density was over 10^7^ cells mL^−1^ while the initial silicate was 3% (w/v) (Fig. 6). This result confirmed the possibility of further increasing biomass production by optimizing growth conditions. Notably, *A. coffeaeformis* also represents a type of algae that can be easily harvested in large-scale cultures because the cells can settle to the bottom of the vessel within a short period after aeration is stopped, which makes it more cost-effective for commercial applications.

#### Effect of *A. coffeaeformis* as feed additive for fish aquaculture

As discussed above, *A. coffeaeformis* can significantly accumulate lipids under N-deficient conditions. Therefore, we decided to evaluate the effect of induced *A. coffeaeformis* as a feed additive on crucian carp's growth and lipid composition. As shown in Table 4, the whole fish proximate analysis was significantly affected by *A. coffeaeformis* supplementation. The results showed a decrease in lipid content (*p* < 0.05) in crucian carp fed with *A. coffeaeformis*, suggesting a positive mobilization of lipids in fish which exhibited lower deposition of lipids [32]. The results also indicated an increase in carcass ash content (*p* < 0.05). This may be attributed to the higher ash content (55.8%) of *A. coffeaeformis* [33].

**Table 4:** Biochemical composition (%, mean ± SE) of crucian carp (*Carassius auratus*) fed the experimental diets

Group	Moisture	Protein	Lipid	Ash
B0	70.32 ± 0.16	18.21 ± 0.28	8.05 ± 0.21	3.42 ± 0.11
B1	70.66 ± 0.23	18.03 ± 0.37	7.53 ± 0.13*	3.78 ± 0.07*

Values in the same column with the different superscripts are significantly different (*p < 0.05)

Considering the use of oil-rich *A. coffeaeformis* as a bait additive in this study, we focused on the muscle lipid composition of crucian carp. The fatty acid composition of muscle samples from the dorsal, ventral, and caudal parts of fish is summarized in Table 5 and showed a significant difference affected by *A. coffeaeformis* additives. In particular, the proportion of saturated fatty acids (SFAs, incl. C15:0 and C16:0) and monounsaturated fatty acids (MUFAs, incl. C18:1 and C20:1) decreased, while the proportion of PUFAs (incl. C18:2, C18:3, EPA, and DHA) increased significantly. This result confirmed the potential of *A. coffeaeformis* as feed additive to improve the fatty acid composition of fish. To our best knowledge, despite microalgae having been used as baits for a long time, rare reports have been published concerning the utilization of *A. coffeaeformis* as a feed additive in aquaculture. Several studies have demonstrated the potential of this microalga for disease resistance and growth promotion. For example, Ayoub et al. [34] found that *A. coffeaeformis* has the potential to replace antibiotics in *Oreochromis niloticus* aquaculture and the optimal level of addition is 1–2% of the fish diet. Similarly, Saleh et al. [32] confirmed that *Amphora* can be used as an immunostimulant for increasing disease resistance of *Oreochromis niloticus* under stress conditions. Both our results and these earlier studies confirm the promising application of *A. coffeaeformis* in aquaculture, which will support the commercial development of this species.

**Table 5:** Fatty acid composition (%, mean ± SD) different sampling sites of crucian carp (*Carassius auratus*) fed the experimental diets

Fatty acid	Back		Belly		Tail	
	B0	B1	B0	B1	B0	B1
C14:0	1.86 ± 0.35	1.49 ± 0.08	2.54 ± 0.24	1.70 ± 0.11*	1.73 ± 0.39	1.27 ± 0.11*
C15:0	0.27 ± 0.02	0.21 ± 0.02*	0.41 ± 0.005	0.27 ± 0.005*	0.27 ± 0.03	0.22 ± 0.03
C16:0	26.28 ± 0.49	23.32 ± 1.19*	25.68 ± 1.89	21.21 ± 1.61*	29.88 ± 1.05	25.30 ± 1.08**
C16:1	4.34 ± 0.18	3.53 ± 0.11*	5.71 ± 0.17	4.24 ± 0.16*	4.16 ± 0.22	2.69 ± 0.15*
C17:0	0.43 ± 0.01	0.40 ± 0.01	0.49 ± 0.09	0.31 ± 0.08	0.44 ± 0.13	0.25 ± 0.02
C18:0	9.16 ± 0.49	9.22 ± 0.69	8.15 ± 0.45	8.35 ± 0.37	11.28 ± 0.65	10.84 ± 0.55
C18:1	32.94 ± 1.76	28.55 ± 1.30*	33.73 ± 1.01	29.32 ± 1.03*	28.87 ± 1.76	23.74 ± 1.60*
C18:2	16.15 ± 1.76	22.61 ± 1.61*	12.29 ± 1.96	22.39 ± 2.02*	10.68 ± 2.63	21.35 ± 0.62*
C18:3	0.96 ± 0.08	1.98 ± 0.14*	0.83 ± 0.28	2.35 ± 0.12*	0.61 ± 0.11	1.57 ± 0.22*
C20:0	0.18 ± 0.03	0.18 ± 0.02	0.19 ± 0.03	0.16 ± 0.02	0.22 ± 0.03	0.22 ± 0.02
C20:1	2.10 ± 0.26	1.47 ± 0.15*	2.90 ± 0.17	2.01 ± 0.09*	2.28 ± 0.09	1.56 ± 0.06*
C20:2	0.56 ± 0.01	0.53 ± 0.07	0.52 ± 0.06	0.59 ± 0.05	0.57 ± 0.05	0.35 ± 0.03
C20:3	0.99 ± 0.07	1.01 ± 0.01	0.69 ± 0.19	0.98 ± 0.14	0.93 ± 0.51	0.97 ± 0.04
C20:4	0.17 ± 0.01	0.21 ± 0.04	0.21 ± 0.03	0.20 ± 0.03	0.35 ± 0.03	0.31 ± 0.06
C20:5	1.02 ± 0.12	1.59 ± 0.18*	1.65 ± 0.18	1.86 ± 0.24	1.73 ± 0.03	2.10 ± 0.06*
C22:1	0.18 ± 0.03	0.11 ± 0.01	0.45 ± 0.05	0.27 ± 0.01*	0.10 ± 0.01	0.19 ± 0.02
C22:6	2.41 ± 0.19	3.66 ± 0.19*	3.54 ± 0.17	3.78 ± 0.32	5.16 ± 0.43	7.06 ± 0.36**
∑MUFA	39.56 ± 0.51	33.58 ± 0.36**	42.79 ± 1.63	35.84 ± 0.71*	35.40 ± 0.98	28.18 ± 1.26*
∑PUFA	22.27 ± 1.41	31.59 ± 2.35*	19.74 ± 1.82	32.16 ± 2.99*	20.79 ± 2.37	33.71 ± 3.39*

Values in the same row with the different superscripts are significantly different (*p < 0.05; **p < 0.01)

## Conclusion

In the present study, the mechanism underlying lipid accumulation under N deprivation in *A. coffeaeformis* was investigated by RNA-seq. We found that GPAT may be a rate-limiting step in TAG biosynthesis pathway of *A. coffeaeformis*, which would be a potential target for the improvement of TAG productivity in microalgae. Then, we evaluated the effects of *A. coffeaeformis* as feed supplement on crucian carp. Results demonstrated that the addition of *A. coffeaeformis* resulted in the depletion of fish lipid content and increased the proportion of unsaturated fatty acid in fish muscle.

## Materials and methods

### Microalgal strain and growth condition

The *A. coffeaeformis* strain was collected from the Atlantic coastal zone of Namibia. The purified *A. coffeaeformis* was incubated in Petri dishes containing 20 mL f/2 medium. The culture condition was as follows: 20 °C, 100–110 μmol photons m^−2^ s^−1^, 12 h/12 h light:dark photoperiod, and 35% salinity. To impose N deficiency stress, the liquid cultures were discarded, and the microalgae attached to the petri dishes were washed thrice with f/2-N medium and finally re-cultured in f/2-N medium.

Amplified culture of *A. coffeaeformis* and optimization of conditions was carried out in a 20-L tubular photobioreactor. The reactor passed filtered air at a flow rate of 1.3 m^3^ min^−1^. To investigate the effects of initial silicate on the growth of *A. coffeaeformis*, f/2 medium containing 1%, 3%, 5%, and 7% silicate was prepared. The other conditions, including temperature, light, and salinity, were not changed.

### Examination of photosystem II activity

To monitor the physical status of *A. coffeaeformis* during N deprivation, the photosynthetic activity as the main representative physical character was measured every 24 h using Water PAM (WALZ, Germany), while *A. coffeaeformis* cultures in normal f/2 medium were set as the positive control. The model of slow light curve was used to test the Y II (Fv/Fm), and Etr. Using different settings, the *A. coffeaeformis* cultures were measured with optimal slow light curve with the following characters. The algae were dark adapted for 10 min to determine the minimal level of fluorescence (F_0_) and the maximal fluorescence (F_m_) after a saturating flash (0.8 s; 5640 μmol m^−2^ s^−1^), and the ratio of variable to maximal fluorescence (Fv/Fm) was calculated. Following a delay of 40 s, the algae were exposed to an actinic illumination of 322 mol m^−2^ s^−1^ for 0.8 s every 20 s during the 5 min width.

### Microscopic observation

To assess the growth cure of *A. coffeaeformis*, single alga cells were separated using capillaries and monitored using an inverted microscope (200 × magnification) every 24 h to count the cell number. During N deprivation, the *A. coffeaeformis* cultures were assessed microscopically for community structures and attachment status.

### Lipid extraction and fatty acid analysis

Approximately 100 mg dry sample was used for total lipid extraction using the ultrasonication and chloroform/methyl alcohol method [35]. Lipid was detected using iodine vapor and quantified using the assay balance. The fatty acid composition was determined using gas chromatography/mass spectrometry as described previously [3].

### Transcriptomic analysis

#### RNA-seq method

After 5 days of N deprivation, total RNA was extracted from three replicates of N-deprived *A. coffeaeformis* and the control using RNaiso (TAKARA, Dalian, China). The RNA samples were treated with RNase-free DNase I (TAKARA, Dalian, China) and enriched with oligo (dT) magnetic beads. The resulting RNA samples were sequenced by the Novogene company (Beijing, China) using a HiSeq™ 2000 (Illumina) instrument. Owing to the lack of genome sequence, *A. coffeaeformis* RNA was analyzed according to the model diatom *P. tricornutum*.

#### Differential expression analysis

Differential expression analysis was performed using the DESeq R package (1.10.1). DESeq provides statistical routines for determining differential expression in digital gene expression data using a model based on the negative binomial distribution. The resulting *p* values were adjusted using the Benjamini and Hochberg’s approach for controlling the false discovery rate. Genes with adjusted *p* values < 0.05 found by DESeq were assigned as differentially expressed. Kyoto Encyclopedia of Genes and Genomes (KEGG) functional enrichment analysis were performed using KEGG Pathway Database (https://www.genome.jp/kegg/pathway.html) by KOBAS software.

#### RNA-seq validation by quantitative real-time PCR

RNA samples (800 ng ~ 1 μg) were used for first strand cDNA synthesis using *Evo M-MLV* RT Premix for qPCR AG11706 (Accurate Biology, Changsha, China). The cDNA concentrations were then determined using 7500 Fast real-time PCR system (Thermo Fisher Scientific, USA) and SYBR Green I detection reagent. The β-actin was as a housekeeping marker for normalization of real-time PCR data.

### Fishes feeding experiment

*A. coffeaeformis* grown in the photoreactor were separated from the culture by auto-sedimentation and then cleaned by sequenced water washing. After the cells were resuspended and incubated in f/2-N medium for 5 days, the biomass was recovered and freeze-dried. The experimental diets were assigned as control [B0, amphora-free added diet, i.e., 100% commercial diet (TASA, Peru)] and examined diet containing 5% dry biomass of *A. coffeaeformis* (B1). The crucian carps (*Carassius auratus*) with average body weight of ca. 10 g were randomly divided into two groups (20 crucian carps per group in triplicate) and fed with these two diets after adapting for 1 week to the experimental conditions. The test diets were given twice daily at 10:00 and 15:00 h to visual satiation. The natural photoperiod was 12-h light:12-h dark throughout the feeding experiment and the water temperature was 21 ± 1.4℃. After a 14-day feeding trial, muscle samples were taken from the dorsal, ventral, and caudal parts of crucian carp and stored at − 20 °C after freeze-drying and grinding until use. Proximate analyses were performed as described by Saleh et al. [32]. Fish maintenance and experimental procedures were approved by the School of Pharmacy, Binzhou Medical University, China, and were in accordance with the Guide for Use and Care of Laboratory Animals (European Communities Council Directive 2010/63/EU).

### Statistical analysis

Statistical analyses were performed using the SPSS statistical package. Biological triplicates were used for all experiments to ensure reproducibility. The experimental data were expressed as the mean ± standard deviation (± SD, *n* = 3). The data of biochemical composition of crucian carp were expressed as the mean ± standard error (± SE, *n* = 3).

### Supplementary Information

**Additional file 1: Fig. S1.** The volcano plot of DEGs in *A. coffeaeformis* under N deprivation. **Fig. S2.** KEGG classification of the assembled transcripts. **Table S1.** Fold changes in the expression of some genes encoding enzymes involved in various metabolisms following N deprivation.

## Data Availability

Data will be made available on request. The transcriptome raw data are available in the SRP database (Access No. PRJNA753251).
