# Supplementary material for: Lipid accumulation mechanism of Amphora coffeaeformis under nitrogen deprivation and its application as a feed additive in Carassius auratus aquaculture
Source: Biotechnol Biofuels Bioprod. 2023 Dec 6;16:189. doi: 10.1186/s13068-023-02436-7 (PMC10702062; doi:10.1186/s13068-023-02436-7)
Supplement: Supplementary file 1 — Additional file 1: Fig. S1. The volcano plot of DEGs in A. coffeaeformis under N deprivation. Fig. S2. KEGG classification of the assembled transcripts. Table S1. Fold changes in the expression of some genes encoding enzymes involved in various metabolisms following N deprivation. [file 13068_2023_2436_MOESM1_ESM.docx]

**Additional file 1**

**Lipid accumulation mechanism of *Amphora coffeaeformis* under nitrogen deprivation and its application as a feed additive in *Carassius auratus* aquaculture**

Yulin Cui ^a, 1^, Kang Wang ^b, c, 1^, Xiuzhi Zhou ^a, 1^, Chunxiao Meng ^a^, Zhengquan Gao ^a*^

^a^ Binzhou Medical University, Yantai, 256603, Shandong, China

^b^ Key Laboratory of Coastal Biology and Biological Resource Utilization, Yantai Institute of Coastal Zone Research, Chinese Academy of Sciences, Yantai, 264003, Shandong, China

^c^ University of Chinese Academy of Sciences, Beijing, 101418, Beijing, China

*Correspondence:

Zhengquan Gao, email: gaozhengquan@bzmc.edu.cn, tel: 0086 533 2762265. No. 346, Guanhai Road, Laishan District, Yantai, Shandong Province, China.

^1^ These authors contributed equally to this work and are co-first authors.

**Fig. S1. The volcano plot of DEGs in *A. coffeaeformis* under N deprivation.**

**
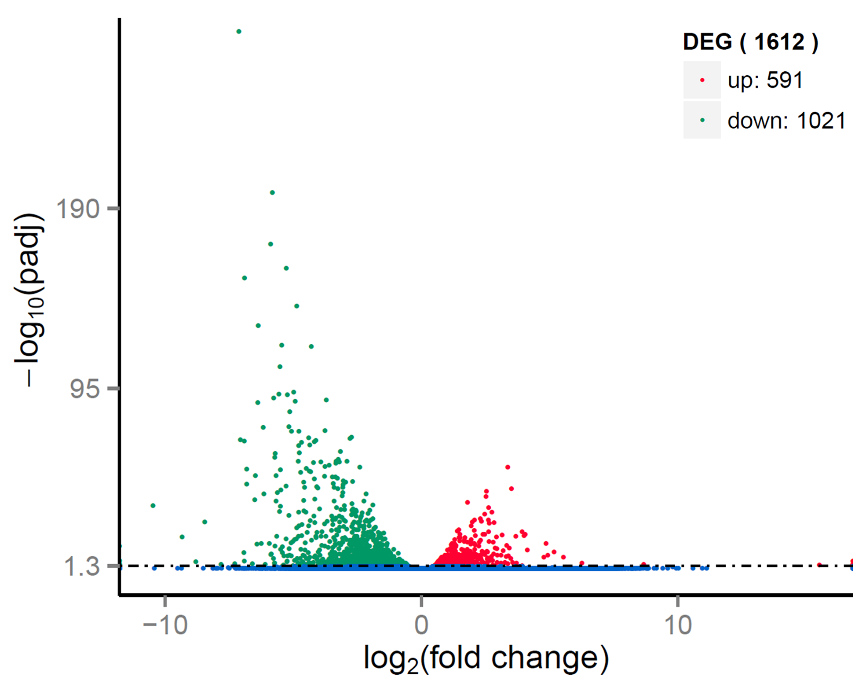
**

**Fig. S2. KEGG classification of the assembled transcripts.** A, cellular processes; B, environmental information processing; C, genetic information processing; D, general metabolism; E, organismal system.


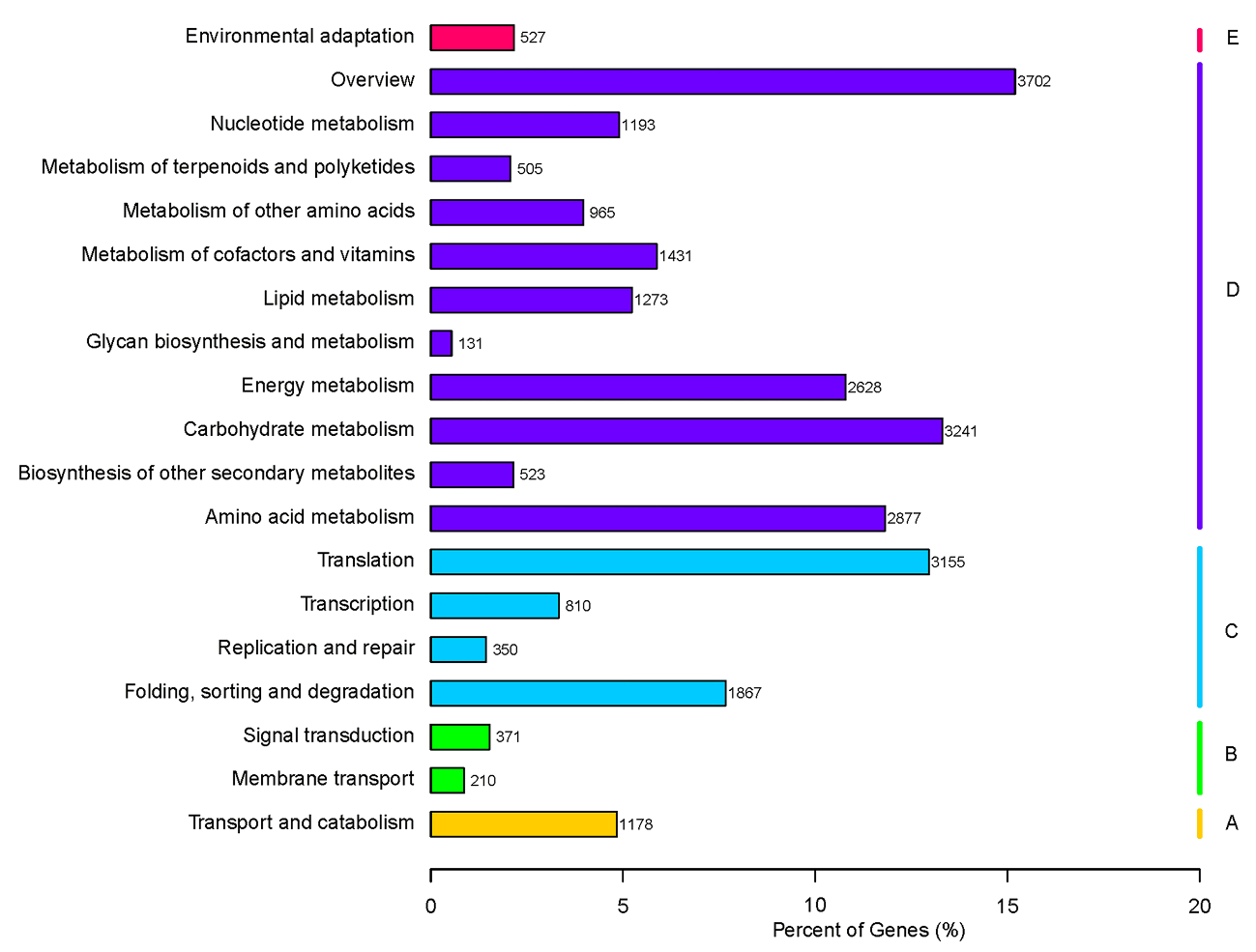


**Table S1. Fold changes in the expression of some genes encoding enzymes involved in various metabolisms following N deprivation.**

| **Gene ID** | **Annotation** | **-N (RC)** | **Ctr (RC)** | **Log2 (-N/Ctr)** |
| --- | --- | --- | --- | --- |
| **a) Nitrogen metabolism** | | | | |
| 15275 | nitrate reductase | 372.514988 | 35.930089 | 3.374 |
| 18580 | glutamine synthetase | 7599.86143 | 2620.131229 | 1.5363 |
| 22850 | glutamine synthetase | 5649.566353 | 1314.546504 | 2.1036 |
| 18662 | glutamate synthase | 2631.326721 | 896.006541 | 1.5542 |
| 21656 | glutamate synthase | 4592.013453 | 1648.15029 | 1.4783 |
| 3059 | nitrate/nitrite transporter | 522.5357863 | 14.41350449 | 5.18 |
| 18218 | nitrate/nitrite transporter | 819.1701596 | 1985.631475 | -1.2774 |
| 21159 | nitrate/nitrite transporter | 2022.101247 | 4786.59297 | -1.2431 |
| 24287 | nitrate/nitrite transporter | 2483.158466 | 230.8547903 | 3.4271 |
| 24803 | nitrate/nitrite transporter | 2205.7077 | 858.0521102 | 1.3621 |
| **b) Photosynthesis** | | | | |
| 16006 | ferredoxin-NADP^+^ reductase | 6524.733707 | 406.6887765 | 4.0039 |
| 19823 | ferredoxin-NADP^+^ reductase | 287.4204597 | 1141.620768 | -1.9898 |
| 21017 | ferredoxin-NADP^+^ reductase | 229.6194488 | 6308.061541 | -4.7799 |
| 21238 | plastocyanin reductase | 3733.492622 | 8923.667631 | -1.2571 |
| 20855 | cytochrome c6 | 7139.358508 | 49285.20064 | -2.7873 |
| 20414 | photosystem II oxygen-evolving enhancer protein | 2423.687243 | 70912.05802 | -4.8708 |
| 21545 | photosystem II PsbU protein | 6157.476445 | 67048.99892 | -3.4448 |
| 22299 | photosystem II PsbU protein | 73.26160807 | 1089.099582 | -3.8939 |
| 18665 | photosystem II Psb27 protein | 202.1556032 | 1402.065782 | -2.794 |
| 18089 | chlorophyll a/b binding protein | 103.2725483 | 480.6728639 | -2.2186 |
| 19066 | chlorophyll a/b binding protein | 101.9006798 | 8413.258399 | -6.3674 |
| 19875 | chlorophyll a/b binding protein | 82.22104486 | 9849.406111 | -6.9044 |
| 20597 | chlorophyll a/b binding protein | 3160.505973 | 19506.54674 | -2.6257 |
| 20721 | chlorophyll a/b binding protein | 1567.779659 | 37620.59396 | -4.5847 |
| 21274 | chlorophyll a/b binding protein | 593.9858573 | 22999.15866 | -5.275 |
| 21440 | chlorophyll a/b binding protein | 2100.489624 | 31932.96318 | -3.9262 |
| 21499 | chlorophyll a/b binding protein | 169.051626 | 789.8286091 | -2.2241 |
| 21898 | chlorophyll a/b binding protein | 112.5289614 | 6129.862889 | -5.7675 |
| 21901 | chlorophyll a/b binding protein | 153.8253808 | 11111.98202 | -6.1747 |
| 21957 | chlorophyll a/b binding protein | 305.9026105 | 18132.13828 | -5.8893 |
| **c)** **Carbohydrate metabolism** | | | | |
| 23708 | phosphoglucomutase | 561.9417468 | 1444.103523 | -1.3617 |
| 21418 | glucose-6-phosphate isomerase | 393.2216649 | 2116.930303 | -2.4286 |
| 18528 | 6-phosphofructokinase 1 | 1755.570673 | 3815.9091 | -1.1201 |
| 16698 | fructose-1,6-bisphosphatase I | 34.42486455 | 370.962968 | -3.4298 |
| 20779 | fructose-1,6-bisphosphatase I | 3840.836003 | 9391.592221 | -1.2899 |
| 21261 | fructose-1,6-bisphosphatase I | 715.1654759 | 2849.622213 | -1.9944 |
| 21835 | fructose-bisphosphate aldolase | 1237.234706 | 11721.14528 | -3.2439 |
| 23904 | fructose-bisphosphate aldolase | 567.6812127 | 2403.477382 | -2.082 |
| 26572 | fructose-bisphosphate aldolase | 43.03560487 | 319.2633999 | -2.8911 |
| 19631 | triosephosphate dehydrogenase | 2181.947394 | 10855.91448 | -2.3148 |
| 21116 | triosephosphate dehydrogenase | 41304.70278 | 69435.72308 | -0.74937 |
| 23704 | triosephosphate isomerase | 518.8210027 | 4591.391125 | -3.1456 |
| 18736 | phosphoglycerate kinase | 43.52002363 | 2143.247823 | -5.622 |
| 18957 | phosphoglycerate kinase | 26.22632073 | 592.4165008 | -4.4975 |
| 20382 | phosphoglycerate kinase | 365.3435052 | 1678.931479 | -2.2002 |
| 21480 | phosphoglycerate kinase | 3891.219677 | 12538.01042 | -1.688 |
| 21192 | phosphoglycerate mutase | 153.0356733 | 4299.266489 | -4.8122 |
| 22007 | phosphoglycerate mutase | 803.7777836 | 2054.522339 | -1.3539 |
| 20522 | dihydrolipoamide dehydrogenase | 218.4954806 | 707.7020152 | -1.6955 |
| 19485 | alcohol dehydrogenase | 988.6501276 | 148.9674854 | 2.7305 |
| 21750 | pyruvate kinase | 8376.891093 | 14156.35406 | -0.75696 |
| 21182 | acetyl-CoA synthetase | 15661.34915 | 24816.91536 | -0.66412 |
| 21393 | acetyl-CoA synthetase | 1792.447579 | 5875.072001 | -1.7127 |
| 19613 | glucokinase | 399.0938824 | 1200.363751 | -1.5887 |
| 23839 | phosphoenolpyruvate carboxylase | 2170.954726 | 751.8670532 | 1.5298 |
| 20062 | phosphoenolpyruvate carboxylase | 10259.20994 | 3722.869952 | 1.4624 |
| 18233 | pyruvate-phosphate dikinase | 230.0535757 | 1065.829123 | -2.2119 |
| 19460 | pyruvate-phosphate dikinase | 849.6122 | 2764.841783 | -1.7023 |
| 23338 | alanine transaminase | 441.2485027 | 1123.599286 | -1.3485 |
| 14846 | malate dehydrogenase | 1141.856736 | 161.4788565 | 2.822 |
| 18055 | ribulose-bisphosphate carboxylase | 142.509097 | 715.6545376 | -2.3282 |
| 23261 | phosphoribulokinase | 330.5398251 | 3342.298766 | -3.3379 |
| 14197 | ribose-5-phosphate isomerase | 95.87443166 | 807.1236265 | -3.0736 |
| 20807 | transketolase | 1878.252859 | 7453.940962 | -1.9886 |
| 21374 | bisphosphatase | 246.7051709 | 1546.079706 | -2.6478 |
| **d) TCA cycle** | | | | |
| 21087 | phosphoenolpyruvate carboxykinase | 1886.406417 | 16908.97358 | -3.1641 |
| 22504 | pyruvate carboxylase | 474.4214437 | 3868.092124 | -3.0274 |
| 21934 | aconitate hydratase | 5256.334744 | 2975.154212 | 0.82109 |
| 21779 | succinyl-CoA synthetase subunit | 11724.29314 | 24882.11034 | -1.0856 |
| 19140 | succinate dehydrogenase subunit | 1363.423445 | 3021.180877 | -1.1479 |
| 20540 | succinate dehydrogenase subunit | 2050.685293 | 9391.02257 | -2.1952 |
| **e) Glycerolipid metabolism** | | | | |
| 21874 | aldehyde reductase | 491.8090271 | 1357.935003 | -1.4652 |
| 20498 | glycerophosphate acyltransferase | 7211.230526 | 3049.304318 | 1.2418 |
| 18970 | lysophosphatidic acid-acyltransferase | 1013.096014 | 2302.297269 | -1.1843 |
| 20296 | phospholipid: diacylglycerol acyltra-nsferase | 1447.925401 | 4931.902112 | -1.7682 |
| 21288 | digalactosyldiacylglycerol synthase | 4552.832176 | 1833.257131 | 1.3124 |

Ctr, control; RC, read count.
